# Response2covid19, a dataset of governments’ responses to COVID-19 all around the world

**DOI:** 10.1038/s41597-020-00757-y

**Published:** 2020-11-25

**Authors:** Simon Porcher

**Affiliations:** IAE Paris, Sorbonne Business School, Université Paris I Panthéon-Sorbonne, Paris, 8 bis rue croix de jarry, 75013 Paris, France

**Keywords:** Politics, Government

## Abstract

Following the COVID-19 outbreak, governments all around the world have implemented public health and economic measures to contain the spread of the virus and to support the economy. Public health measures include domestic lockdown, school closures and bans on mass gatherings among others. Economic measures cover wage support, cash transfers, interest rates cuts, tax cuts and delays, and support to exporters or importers. This paper introduces ‘Response2covid19’, a living dataset of governments’ responses to COVID-19. The dataset codes the various policy interventions with their dates at the country-level for more than 200 countries from January 1 to October 1, 2020 and is updated every month. The production of detailed data on the measures taken by governments can help generate robust evidence to support public health and economic decision making.

## Abstract (summary)

**Table:** 

Measurement(s)	prevention and control intervention • Intervention or Procedure • rigidity of public health interventions to face COVID-19 • intensity of economic interventions to face COVID-19
Technology Type(s)	digital curation • Data Collection
Factor Type(s)	rigidity of public health interventions • intensity of economic interventions
Sample Characteristic - Location	Global

Machine-accessible metadata file describing the reported data: 10.6084/m9.figshare.13042139

## Background & Summary

In December 2019, a new coronavirus appeared in Wuhan, China and spread to nearly every country in the first quarter of 2020. In the beginning of November 2020, according to the Johns Hopkins Coronavirus Resource Center (https://coronavirus.jhu.edu/), there were more than 50.25 million confirmed cases and over 1.25 million deaths linked to the virus. The pandemic forced governments all around the world to adopt diverse public health policies and economic measures that are quite unique in history. Public policies data is needed in pandemics to best monitor the spread of infection, but also to understand the diversity in governments’ responses. In order to provide accurate and openly available data, we collected data from other datasets or countries case studies on thirteen public health policies and seven economic measures, at the country-level and on a daily basis, and merged it with the data on daily cases and deaths from the European Center for Disease Prevention and Control (https://www.ecdc.europa.eu/en/geographical-distribution-2019-ncov-cases). The resulting dataset, Response2covid19, tracks the implementation and intensity of 20 governments measures taken during the spread of the pandemic, and is updated on a monthly basis.

We make sense of the coded measures by creating two novel indices of governments’ interventions against COVID-19. The first index measures the rigidity of governments’ responses to COVID-19 and is based on the implementation of thirteen public health measures. The second index quantifies the economic responses to COVID-19 based on the coding of seven types of economic interventions to face the economic downturn following the various public health measures. The indices capture the rigidity of governments’ responses to the pandemic between January 1, 2020, and October 1, 2020 on a country-daily basis. In its current version, the final dataset is made of 62,700 country-day observations. 228 countries are included in the database. The two indices allow cross-country comparisons and the documentation of dynamics within countries.

The dataset is of interest for epidemiologists wishing to link governments’ measures worldwide with the evolution of the number of cases^1^. Several studies already assess the impact of travel restrictions^2^, human mobility restrictions^3^ or various transmission control measures^4^. The dataset is also of interest for social scientists wishing to study the impact of other factors, e.g. democracy or institutions, on the rigidity and the timing of the measures taken. Finally, the coding of the economic measures is also useful to relate economic interventions with economic outcomes such as the gross domestic product or national financial market indices.

### Contextualizing the project

The creation of the dataset presented in this paper can be related to the effort of other academics to map governments’ responses to the pandemic. Hale *et al*.^5^ built the Oxford COVID-19 Government Response Tracker (OxCGRT), a dataset of governments’ responses (www.bsg.ox.ac.uk/covidtracker) that includes different variables related to public health, economic interventions, public campaigns and research incentives for a vaccine. The data collection is based on news articles and government press releases and briefings. Their great and concomitant work leads to the creation of a stringency index from 0 to 100 for each country. Cheng *et al*.^6^ created a government policy activity index ranging from 0 to 100 (https://www.coronanet-project.org/). The authors use an artificial intelligence company to scrap the press related to COVID-19 and to extract some information on the dates and scales of implementation of a wide range of public health measures, e.g. school or restaurant closures, but also mobilizations of volunteers, nurses and doctors. Research assistants then confront the results from the scrapping to web sources and manually coded the items in the CoronaNet dataset. Zheng *et al*.^7^ created the HIT-COVID tracker of 21 non-pharmaceutical interventions in 142 countries, structured in two levels, national or regional. They based their data collection on official sources and secondary sources such as media reports. They use two levels of interventions, partial or strong. Finally, Desvars-Larrive *et al*.^8^ coded a structured dataset of non-pharmaceutical interventions divided in 8 themes and 63 categories in 56 countries, the Complexity Science Hub COVID-19 Control Strategies List (CCCSL). They based their data collection on public health institutions, official government sources, peer-reviewed and non-peer reviewed scientific papers, press releases, newspapers articles and social media. The supplementary information 1 gives detailed information on the differences between the various indicators and provides some comparisons on key measures.

The dataset can also be related to datasets tracking more specifically government interventions in the economy^5,9^. An example is Elgin *et al*.^9^ who created a static index of economic policies to respond to COVID-19, by collecting information on the range of governments’ fiscal and monetary interventions in the end of March 2020.

The Response2covid19 dataset complements the efforts made by other researchers in different manners. First, the public health and economic measures considered are not exactly the same. Our dataset considers for example elections and enhanced surveillance as key variables, which are to the best of our knowledge not considered by the abovementioned datasets. Elections are an important moment in democracies, and postponing elections might be interpreted as the will of the governing power to influence the results of elections. As countries are reopening the economy, mobile app or bracelet surveillance of contaminated individuals is increasingly required to avoid new lockdowns. These measures raise issues in terms of human rights. Second, our dataset covers economic measures to a larger extent than Hale *et al*.^5^ We identify the implementation of six different fiscal policies and the change in the main policy rate. Our typology of economic policies can be used to specifically track the impact of cash transfers and wage support on consumption, or the relationship between credit schemes and companies’ investments. Third, Response2covid19 covers 201 countries on public health measures and 198 on economic measures. It covers a wide range of countries. Only CoronaNet tracks interventions in so many countries.

The resulting dataset provides some value added regarding its sources. First, it translates in a unique and harmonized framework – no measures or just recommendations, partial / regional scale or strict / national scale – the efforts made by our sources to track public health and economic policies implemented during the spread of the pandemic. Second, the variety of sources allows covering a larger number of countries or policies than each source taken individually would do.

## Methods

### Public health measures taken by governments

The coding of public health measures is based on cross-country information reported by the Assessment Capacities Project (ACAPS; https://www.acaps.org/covid19-government-measures-dataset), the International Institute for Democracy and Electoral Assistance (IDEA; https://www.idea.int/publications/catalogue/elections-and-covid-19) for elections, the International Monetary Fund (IMF; https://www.imf.org/en/Topics/imf-and-covid19/Policy-Responses-to-COVID-19) and the United Nations Educational Scientific and Cultural Organization (UNESCO; https://en.unesco.org/covid19/educationresponse) for schools closures. The main source of information is the ACAPS. Their dataset is particularly useful in providing information on both the scale and the dates of the measures implemented. The IMF tracker lists all the public health measures taken at the country-level and is particularly helpful to cross-check the information provided by ACAPS. IDEA lists elections postponed or maintained all around the world since the spread of COVID-19. UNESCO provides a worldwide dataset of school closures all around the world.

Thirteen public health measures were considered - bans on mass gatherings, bans on sporting and recreational events, restaurant and bar closures, domestic lockdowns, international travel restrictions, domestic travel restrictions, curfew, declarations of states of emergency, public testing, enhanced surveillance, obligations to wear masks in the public space, school closures, and the postponement of elections. Each measure is coded 1 or 0, depending on whether it was implemented or not, and as a missing variable if the country is not covered. Measures are coded on a daily basis. Before the implementation, the measure is coded 0; from the day of the implementation and until it is lifted, the measure is coded 1.

The first eleven measures were manually coded from the ACAPS dataset. The format of their dataset does not allow us to directly merge their dataset with another one, as they textually report the measures implemented or discussed. Their dataset requires some reading to qualify whether, and at which scale, a given measure is implemented. School closures were directly taken from the UNESCO dataset which indicates on which scale schools are closed (local level, national-level or no school closures). The dataset was simply merged with our baseline dataset. Finally, the IDEA simply reports the postponed or maintained elections with their dates. In the latter case, the coding was done manually.

Additionally, extra variables which equal 1 if the implementation of a given measure is partial or localized, and 0 if it is strict or national, were added. This coding allows researchers to differentiate the degrees of implementation of the measures. The following differentiations were made.

- Bans on mass gatherings, if the ban was localized, the measure was considered partial;
- Cancellation of sporting and large events, if the cancellation was localized, targeted at some events or events could occur with a limited number of persons, the measure was considered partial;
- Travel restrictions, if international commercial flights are still allowed, except to or from some countries, with recommendations to avoid all non-essential travel, the measure was considered partial;
- Domestic travel restrictions, if domestic commercial travels are still allowed, except to or from some regions, with recommendations to avoid all non-essential travel, the measure was considered partial;
- Domestic lockdowns, the measure is considered partial if the domestic lockdowns occurred only in a localized area of the country;
- Curfew, if curfews occurred only in a localized area of the country, the measure is considered partial;
- Restaurant and bar closures, when the closures were localized and/or the ban limited, e.g. a limited number of clients can still sit in the restaurants, the measure was considered partial;
- Mandates to wear masks, when the obligations to wear masks in public spaces is localized, the measure was considered partial;
- Public testing, when the public testing policy was targeted to certain categories of the population (e.g. health personnel or teachers), it was considered partial;
- Enhanced surveillance, enhanced surveillance is considered partial if mobile app or bracelet surveillance is optional or targeted towards a given public (e.g. foreigners). It is coded as strict if their use is compulsory for individuals who tested positive or for the whole population;
- State of emergency, the measure is considered partial if the state of emergency is declared at the local level;
- Elections, we followed IDEA which reports whether all elections were postponed (strict) or only some elections were postponed and some others maintained (partial);
- Schools, we followed the UNESCO which reports whether the closures are localized or national.

The coding of the dataset is based on two binary flags indicating the implementation of the measures, and whether measures are partial or strict. The two binary variables can be crossed to get an ordinal scale of the implementation of a given measure (e.g. 0 = no measure, 0.5 = partial measure and 1 = strict measure). Such a scale is useful to reduce the potentiality of measurement error and increases the reliability of the coding.

**Fig. 1:**
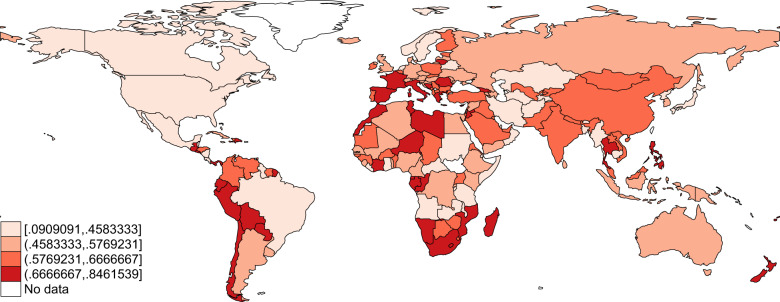
Static map of the index of public health responses to COVID-19 as of April 15, 2020. The index ranges between 0 and 1, with 0 being the lowest possible value (no public health response to COVID-19) and 1 being the highest value (all potential listed policies are implemented at the national-level).

**Fig. 2:**
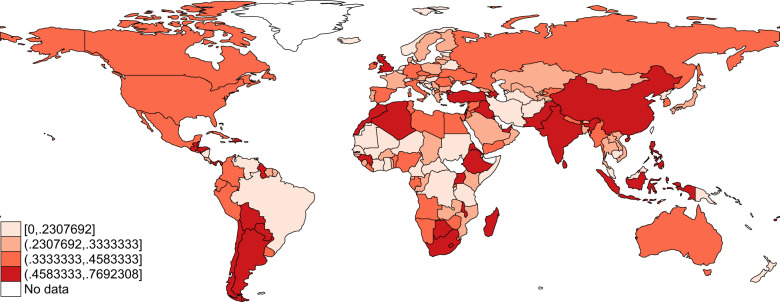
Static map of the index of public health responses to COVID-19 as of September 15, 2020. The index ranges between 0 and 1, with 0 being the lowest possible value (no public health response to COVID-19) and 1 being the highest value (all potential listed policies are implemented at the national-level).

The thirteen public health measures can be used to create an index of the rigidity of governments’ public health responses to COVID-19. While the scale of the measures is limited to three possibilities, the number of measures allows for a certain granularity of the indexes. Figures 1 and 2 are static maps of the rigidity index on April 15, 2020 and September 15, 2020, all around the world. To build the index represented in the figures, we recoded, for each measure, strict and/or national measures as 1, partial and/or localized measures as 0.5 and no measures as 0. The index of rigidity is the mean of the coded indicators and ranges between 0 and 1. The index of rigidity is computed for observations with at least 10 indicators fulfilled out of 13. If there are more than 3 missing indicators, the index of Rigidity is not computed and considered as missing.

### Economic measures taken by governments

**Fig. 3:**
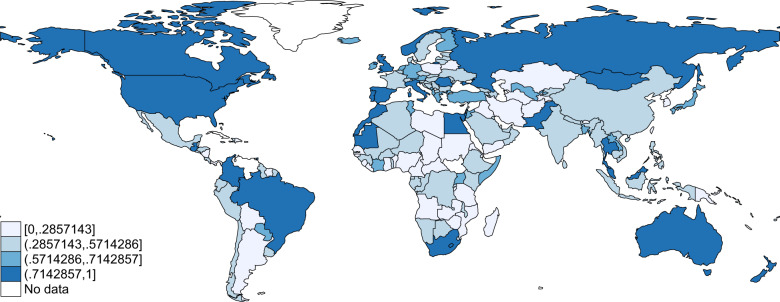
Static map of the index of economic responses to Covid-19 as of April 15, 2020. The index ranges between 0 and 1, with 0 being the lowest possible value (none of the considered economic responses to COVID-19 is implemented) and 1 being the highest value (all potential listed economic measures are implemented).

**Fig. 4:**
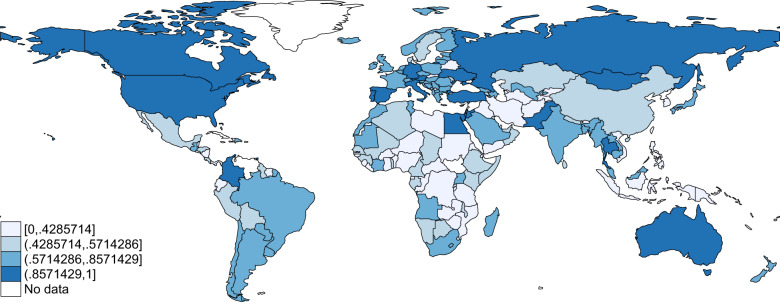
Static map of the index of economic responses to Covid-19 as of September 15, 2020. The index ranges between 0 and 1, with 0 being the lowest possible value (none of the considered economic responses to COVID-19 is implemented) and 1 being the highest value (all potential listed economic measures are implemented).

Another index is based on the coding of economic measures taken to face the economic downturn following public health measures of containment. The information comes from the International Monetary Fund (IMF) and the International Growth Centre (IGC; https://www.theigc.org/covid-19/tracker/). For each country we read the list of measures listed by the IMF and followed the IGC in the creation of seven categories of economic intervention - wage support, cash transfer, credit schemes, tax cuts and delays, support to importers and exporters, and policy rate cuts. The coding is done on a binary basis, 0 or 1, depending on whether the measure is implemented or not. As for public health measures, the coding is done a country-daily basis. The day of the announcement is either reported in the country-profile of the IMF or in the list of economic measures of the ACAPS. The index of economic measures is the mean of the seven variables coded and ranges between 0 and 1. Figures 3 and 4 are static maps of the index of economic measures on April 15, 2020 and September 15, 2020 all around the world.

**Fig. 5:**
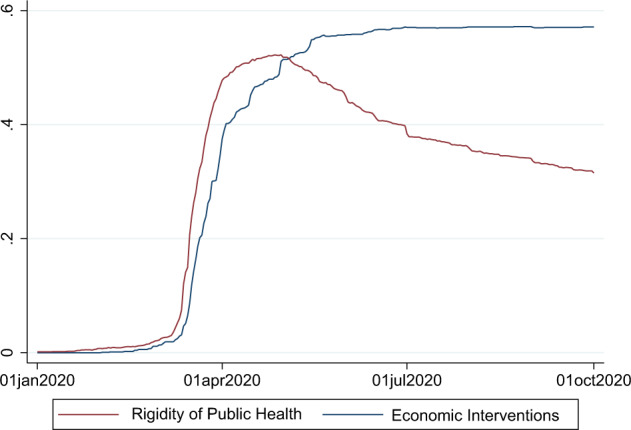
Evolution of the global index of the rigidity of public health responses and the global index of economic responses. The global indexes shows that economic interventions come in a second step to face COVID-19 but become more intense as the situation evolves.

The index of rigidity of public health interventions and the index of economic measures can be plotted for each country or for the world. Figure 5 shows the evolution of the two indexes at the global level from January to October 2020.

### Custom codes used in the work

For all variables, except the variable school closures which is in a suitable dataset format by the UNESCO, the coding of the data is done directly in a Stata do file via lines of coding. Each line of coding identifies the measure to be coded, the scale of the measure, the targeted country and the period for which the measure is implemented. The format of the coding is standard and easy to read for non-Stata users. For example, the national domestic lockdown in France started on March 17 and was lifted on May 11. The line coded is *replace domestic* = *”Strict” if iso* = *”FRA” & tin(17mar2020, 10may2020)*. The spreadsheet data is created from the coding script, available on the online repository (“script.do”). There are many advantages to use such a methodology, instead of writing directly in the spreadsheet.

1. It prevents the coder from entering a given measure at the wrong date or for the wrong country. Errors on measures, scales, dates and countries are more visible to the cross-checkers than in a spreadsheet format;
2. It reduces missing data as it forces the coders to fulfill the dates of implementation of the policies. It also gives more valuable knowledge of the dataset as we observe the changes in the intensity of the measures across time and the timing of the events;
3. When the coding script is run, it indicates for each line the number of changes written in the dataset. No changes indicated flag an issue in the coded line.

## Data Records

We have established a Github repository (https://github.com/simonporcher/COVID-19-Governments-Responses) and an Open ICPSR repository^10^ where new data is uploaded every month. The updated versions of the database can be downloaded from GitHub or OpenICPSR in Excel and Stata formats and can be imported into a variety of software programs. The file “Sources.xls” in the repository reports, for each country, lists the sources used for the economic and public health measures. Data visualizations are available at https://response2covid19.org/.

As the situation regarding the COVID-19 outbreak is continuously evolving, the repositories list the former versions of the datasets with the Stata do files (script.do) tracking the updated changes for each version.

Each row in the database represents the situation in a country at a given date between January 1, 2020 and October 1, 2020. A description of the fields in the database is shown below and is available through a data dictionary on the repositories.

- ***country***, name of the country or the territory;
- ***geoid***, two-letters country code;
- ***iso***, three-letters country code;
- ***d***, date of the observation;
- ***cases****,* number of cases reported on the given day by the European Centre for Disease Prevention and Control;
- ***deaths****,* number of deaths reported on the given day by the European Centre for Disease Prevention and Control;
- ***school***, binary variable equal to1 if schools were closed and 0 otherwise;
- ***school_local****,* binary flag to distinguish localized school closures from other cases. 1 denotes that school closures were implemented at the local level and 0 denotes that school closures were not implemented at the local level (either at the national level or no school closures). The data on the scale of school closures is imported from the UNESCO. The interaction of *school* and *school_local* allows researchers to create three levels of measures, no school closures (*school* = 0 and *school_local* = 0), localized school closures (*school* = 1 and *school_local* = 1) or national school closures (*school* = 1 and *school_local* = 0);
- ***domestic***, binary variable equal to 1 if there was a domestic lockdown and 0 otherwise;
- ***domestic_local***, binary variable to distinguish localized domestic lockdowns from other cases. 1 denotes that domestic lockdowns were implemented at the local level and 0 means that domestic lockdowns were not implemented at the local-level (either at the national level or not implemented). The nature of the domestic lockdown is based on our reading of the measures reported by the ACAPS. The interaction of *domestic* and *domestic_local* allows researchers to create three levels of measures, no domestic lockdown (*domestic* = 0 and *domestic_local* = 0), localized domestic lockdowns (*domestic* = 1 and *domestic_local* = 1) or national domestic lockdowns (*domestic* = 1 and *domestic_local* = 0);
- ***travel***, binary variable equal to1 if travel restrictions were implemented and 0 otherwise;
- ***travel_partial***, binary flag to differentiate partial travel restrictions from other cases. 1 denotes that travel restrictions were partial and 0 denotes that travel restrictions were not partial (either strict or not implemented). The nature of the travel restrictions is based on our reading of the measures reported by the ACAPS. The interaction of *travel* and *travel_partial* allows researchers to create three levels of measures, no travel restrictions (*travel* = 0 and *travel_partial* = 0), partial travel restrictions (*travel* = 1 and *travel_partial* = 1) or strict travel restrictions (*travel* = 1 and *travel_partial* = 0);
- ***travel_dom***, binary variable equal to1 if travel restrictions within the country (e.g. inter-region travels) were implemented and 0 otherwise;
- ***travel_dom_partial***, binary flag to differentiate partial domestic travel restrictions from other cases. 1 denotes that travel restrictions were partial and 0 denotes that domestic travel restrictions were not partial (either strict or not implemented). The nature of the travel restrictions is based on our reading of the measures reported by the ACAPS. The interaction of *travel* and *travel_partial* allows researchers to create three levels of measures, no domestic travel restrictions (*travel_dom* = 0 and *travel_dom_partial* = 0), partial domestic travel restrictions (*travel_dom* = 1 and *travel_dom_partial* = 1) or strict domestic travel restrictions (*travel_dom* = 1 and *travel_dom_partial* = 0);
- ***curf****,* binary variable equal to1 if a curfew was implemented and 0 otherwise;
- ***curf_partial***, binary flag to differentiate partial curfews from other cases. 1 denotes that the curfew was partial and 0 denotes that the curfew was not partial (either strict or not implemented). The nature of the curfew is based on our reading of the measures reported by the ACAPS. The interaction of *curf* and *curf_partial* allows researchers to create three levels of measures, no curfew (*curf* = 0 and *curf_partial* = 0), partial curfew (*curf* = 1 and *curf_partial* = 1) or strict curfew (*curf* = 1 and *curf_partial* = 0);
- ***mass***, binary variable equal to1 if bans on mass gatherings were implemented and 0 otherwise;
- ***mass_partial***, binary flag to distinguish localized bans on mass gatherings from other cases. 1 denotes that bans on mass gatherings were partial and 0 denotes that bans on mass gatherings were not partial (either strict or not implemented). The nature of the bans on mass gatherings is based on our reading of the measures reported by the ACAPS. The interaction of *mass* and *mass_partial* allows researchers to create three levels of measures, no bans on mass gatherings (*mass* = 0 and *mass_partial* = 0), localized or partial bans (*mass* = 1 and *mass_partial* = 1) or national or strict bans (*mass* = 1 and *mass_partial* = 0);
- ***elect***, binary variable equal to1 if some elections were postponed and 0 otherwise;
- ***elect_partial***, binary flag to differentiate countries which postponed only some of the elections from the others. 1 denotes that countries both maintained and postponed elections and 0 denotes that elections were either postponed, maintained or were not scheduled. IDEA lists all maintained and postponed elections since the beginning of 2020. The interaction of *elect* and *elect_partial* allows researchers to differentiate three settings, all elections were maintained despite COVID-19 (*elect* = 0 and *elect_partial* = 0), some elections were maintained and others were postponed (*elect* = 1 and *elect_partial* = 1) or all elections were postponed (*elect* = 1 and *elect_partial* = 0);
- ***sport***, binary variable equal to1 if bans on sporting and large events were implemented and 0 otherwise;
- ***sport_partial***, binary flag to distinguish partial bans and cancellations of sporting and large events. 1 denotes that bans on sporting and large events were localized, strict or with no spectators, 0 that bans on sporting and large events are not localized or partial (either national or no measures implemented). The nature of the bans on sporting and large events is based on our reading of the measures reported by the ACAPS. The interaction of *sport* and *sport_partial* allows researchers to create three levels of measures, no bans (*sport* = 0 and *sport_partial* = 0), partial bans (*sport* = 1 and *sport_partial* = 1) or national bans on mass gatherings (*sport* = 1 and *sport_partial* = 0);
- ***rest****,* binary variable equal to1 if restaurants were closed and 0 otherwise;
- ***rest_local***, binary flag to distinguish localized and/or partial restaurant and bar closures from other cases. The variable is coded 1 in the three following situations, localized closures, limitations on the number of customers in bars and restaurants, and closures of either bars or restaurants. 0 indicates national closures or no closures at all. The coding is based on our reading of the measures reported by the ACAPS. The interaction of *rest* and *rest_local* allows researchers to create three levels of measures, no closures (*rest* = 0 and *rest_local* = 0), localized closures (*rest* = 1 and *rest_local* = 1) or national closures (*rest* = 1 and *rest_local* = 0);
- ***testing***, binary variable equal to1 if there was a public testing policy and 0 otherwise;
- ***testing_narrow***, binary flag to distinguish narrow testing policies from large testing policies. 1 denotes that testing policies were targeted to some individuals, 0 that testing policies were not targeted (either large or not implemented). The nature of the testing policy is based on the information reported in the measures “mass population testing” and “testing policy” in the ACAPS. When the measure was targeted, *testing_narrow* was coded 1. On the contrary, when the measure was not targeted, *testing_narrow* was coded 0. The interaction of *testing* and *testing_narrow* allows researchers to create three levels of measures, no testing policy (*testing* = 0 and *testing_narrow* = 0), narrow testing policy (*testing* = 1 and *testing_narrow* = 1) or large testing policy (*testing* = 1 and *testing_narrow* = 0);
- ***surveillance***, binary variable equal to1 if mobile app or bracelet surveillance was implemented and 0 otherwise;
- ***surveillance_partial***, binary variable equal to1 if the enhanced surveillance is optional or reserved for a category of person (e.g. certain professions or foreigners) and 0 otherwise, based on information in the ACAPS. When the measure was partial, *surveillance_partial* was coded 1. On the contrary, when the measure was strict (anybody suspected of having COVID-19 or compulsory for everybody), *surveillance_partial* was coded 0. The interaction of *surveillance* and *surveillance_partial* allows researchers to create three levels of measures, no surveillance (*surveillance* = 0 and *surveillance_partial* = 0), partial surveillance (*surveillance* = *1* and *surveillance_partial* = 1) or strict surveillance (*surveillance* = 1 and *surveillance_partial* = 0);
- ***masks***, binary variable equal to1 if mandates to wear masks in public spaces were implemented and 0 otherwise;
- ***masks_partial***, binary variable equal to1 if the obligation to wear masks is regional, based on information in the ACAPS. When the measure was regional, *masks_partial* was coded 1. On the contrary, when the measure was national, *masks_partial* was coded 0. The interaction of *masks* and *masks_partial* allows researchers to create three levels of measures, no obligations to wear masks (*masks* = 0 and *masks_partial* = 0), regional obligations to wear masks (*masks* = *1* and *masks_partial* = 1) or national obligations to wear masks (*masks* = 1 and *masks_partial* = 0);
- ***state***, binary variable equal to1 if the state of emergency is declared and 0 otherwise;
- ***state_partial***, binary variable equal to1 if the state of emergency is declared on a local basis and 0 otherwise, based on information in the ACAPS. When the measure was local, *state_partial* was coded 1. On the contrary, when the measure was not localized, *state_partial* was coded 0. The interaction of *state* and *state_partial* allows researchers to create three levels of measures, no state of emergency (*state* = 0 and *state_partial* = 0), partial state of emergency (*state* = 1 and *state_partial* = 1) or national state of emergency (*state* = 1 and *state_partial* = 0);
- ***cash***, binary variable equal to1 if cash transfers are implemented and 0 otherwise;
- ***wage****,* binary variable equal to1 if wage support is implemented and 0 otherwise;
- ***credit****,* binary variable equal to1 if credit schemes are implemented and 0 otherwise;
- ***taxc***, binary variable equal to1 if tax credits are implemented and 0 otherwise;
- ***taxd***, binary variable equal to1 if tax delays are implemented and 0 otherwise;
- ***export****,* binary variable equal to1 if supports to importers or exporters are implemented and 0 otherwise;
- ***rate***, binary variable equal to1 if the Central Bank lowered the interest rates and 0 otherwise;
- ***Rigidity_Public_Health***, average of the thirteen coded public health measures. Public health measures are valued 0.5 if they are localized or partial and 1 if they are national or strict. 0 indicates no measures. The index is computed for observations with at least 10 indicators fulfilled out of 13;
- ***Economic_Measures***, average of the seven economic measures.

## Technical Validation

The database was checked on a rolling basis using two complementary methodologies. One was manual and the other was machine enabled.

In the first version of the dataset, the principal investigator manually entered the information in an excel file and exchanged with a research assistant and manually checked the accuracy of the coding of the data for each country on a rolling basis. The dataset was sent via email to the research assistant who checked that the coding was accurate. The checks on the previous versions of the dataset were done on March 30, April 17, 2020, between May 25 and May 29, between June 29 and July 3 and between July 24 and 31, 2020. For economic measures, the research assistant went through the two sources (IGC if the country was coded in their dataset, IMF if the country was not coded in the IGC) and orally listed for each country the economic measures implemented. The principal investigator controlled in the dataset, using the “summarize” function in Stata 14 (https://www.stata.com/), that the coded measures were the same as the ones reported by the sources. When the coding was not accurate, we checked that the policy was not implemented after the last verification in the ACAPS dataset, and updated the dataset with a measure change or modified the mistake.

The same manual methodology was used for public health measures: the research assistant filtered the ACAPS dataset on the different categories of interventions (lockdowns, movement restrictions, public health measures, and social distancing) and orally listed the measures taken at the country-level with their dates. As the ACAPS coding is sometimes anomalous, the research assistant had to go through all the lines for each country when needed. We checked that the coding was accurate using the “tabulate” function in Stata for all coded measures. The tabulate function allows us to summarize information for a given country at a given date. As the ACAPS dataset records the dates of policy measures, modifications were directly made – if necessary – for measures implemented between the two rounds of data verification. The verifications on the first versions of the dataset are listed in the Stata do file “verif.do” in the former version of the dataset in the GitHub repository.

Since the end of June, in order to be swifter in the verification of the different changes, we created a Stata do file which directly codes the spreadsheet from lines of coding (script.do). The script gathers both the former verifications and the first version of the coding which was directly done in the spreadsheet. It allows us to rapidly update dates and scales when measures are extended, relaxed or lifted.

The dataset is now updated every month and the checks are divided in two rounds of manual verifications. The principal investigator coded the potential policy updates in the latest version of the ACAPS dataset between October 15 and October 25, 2020. A research assistant double checked manually, re-reading all the lines reported in ACAPS country per country, that the coded measures were accurate between October 25 and November 6, 2020. A research assistant updated the economic measures reported by the IMF between October 25 and October 30, 2020. The principal investigator manually checked that the coding was accurate, based on his reading of the IMF measures, and did the latest update in the first week of November. In the final check for the economic measures, the public health measures are also cross-checked once again as the IMF now lists all the public health measures implemented at the country-level (without the dates). For the list of democratic elections covered by IDEA, the principal investigator did the secondary check directly on November 5, 2020.

The other verification was made in Stata to check that the coding was consistent before and after the implementation of a given policy, and after the end of the lockdown. To do so, the principal investigator used the “tsline” function in Stata to graph the time series of the indices to check that there was no unexpected break in the tendencies. This graphical check was run for the 37 OECD countries. If the trend was unexpected – e.g. with a decreasing trend at some date or values higher than 0 before any COVID-19 cases was reported – the principal investigator checked that the coding of the measures was accurate and particularly that, at date t-1 before the implementation of a given measure, the coding was 0, and that, at date t + 1, the coding was 1. Potential mistakes were corrected by refereeing to the sources.

## Usage Notes

The dataset codes thirteen public health measures and seven economic policies taken by governments to respond to COVID-19. The coding of the dataset is based on two binary variables for each measure to flag whether it is implemented and the scale of the implementation. We are conscious that it limits the nuances of the policies taken and that it removes part of the information reported in the sources.

The raison d’être of the dataset is not to cross-check that the different sources are identically tagged or to control for the quality of the sources. Such an effort has been undertaken for public health measures by the World Health Organization (WHO; https://www.who.int/emergencies/diseases/novel-coronavirus-2019/phsm). The WHO PHSM resource harmonizes the coding of public health measures listed by a variety of core sources, including ACAPS, and can be leveraged to control the information reported by the core sources. The coding script (script.do) allows researchers and organizations to assess the accuracy of our dataset and the quality of the sources.

We are aware that collecting data from other sources might lead to the importation of any measurement issues they bring with them. The ACAPS dataset provides a full range of information going from political declarations to the detailed implementation of public health or social measures. Our reading of the ACAPS dataset translates the information reported in the ACAPS dataset and scales the selected measures. The IMF gives short case studies of countries but does not provide a dataset format or a list of measures per country, so the quality of the information relies on their treatment of the information and our understanding of the measures. The dataset on schools closures comes from the UNESCO and does not allow differentiating the different levels of schools which are closed.

Users of the dataset must keep in mind that countries do not have the same institutional settings. A federal country like the United States typically gives greater responsibility to state governments to implement national measures and rarely present singular country-level responses. Moreover, the indexes summarizing the different measures should be interpreted as indicators of governments’ reactions to COVID-19. They do not integrate whether the measures were correctly implemented. Furthermore, the level of the indexes should not be interpreted as proxies for good or bad governance.

The dataset is based on manual recording of policy measures implemented all around the world. Even though we made the best attempt to report data as accurately as possible, there might be some remaining errors and we apologize in advance for that. Please email the corresponding author if you wish to point some errors or leave a message on the GitHub repository.

## Supplementary information

Supplementary material

## Data Availability

The coding of the measures is available in a Stata do file format. The author used Stata 14 to produce the final spreadsheet.
